# The regulatory technology “RegTech” and money laundering prevention in Islamic and conventional banking industry

**DOI:** 10.1016/j.heliyon.2020.e04949

**Published:** 2020-10-08

**Authors:** Meaad Turki, Allam Hamdan, Richard Thomas Cummings, Adel Sarea, Magdalena Karolak, Mohammad Anasweh

**Affiliations:** a Dean of College of Business and Finance, Ahlia University, Manama, Bahrain; b Department of Accounting, Finance and Banking, College of Business and Finance, Ahlia University, Manama, Bahrain; c Zayed University, Dubai, United Arab Emirates; d Department of Finance and Banking, School of Business, Mutah University, Jordan

**Keywords:** Regulatory technology (RegTech), Anti money laundering (AML), Know your customer (KYC) compliance, Business management, Business policy, Corporate finance, International finance, Sustainable business

## Abstract

This study aims to illustrate the impact of adopting Regulatory technology (RegTech) innovations in banks on money laundering prevention effectiveness using Bahrain as a case study. Bahrain has strived to position itself as the banking center of the Arabian Gulf, hence the results of this novel research are informative of the practices in the region. The primary data for this study was collected through a survey instrument distributed to 100 bankers working in Bahrain with expertise in compliance. The results of multivariate analysis indicate that transactions monitoring through RegTech and cost- and time-saving aspects of RegTech, drive money laundering prevention effectiveness to a highly statistically significant extent. However, electronic know your customer (KYC) technologies are insignificant as drivers. This research not only sheds light on the efficacy of RegTech but also raises general awareness concerning the adoption and integration of RegTech platforms for fighting money laundering. In particular, the findings provide specific insights about the deployment of RegTech capabilities in banks in regional banking centers of modest scale.

## 1 Introduction

While the coining of the term RegTech in 2015 is commonly credited to the Financial Services Authority (FSA) [EQS (2019)], the UK regulator, the notion underlying this concept began to develop much earlier in the aftermath of regulatory tightening in financial sectors world-wide following the Global Financial Crisis (2008). The landscape of the financial services sector has been gradually changing due to an overhaul in financial regulation but also because of great advances in financial technology innovation (Anagnostopoulos, 2018) after which compliance costs skyrocketed (Hammond and English, 2016). The 2008 financial crisis exposed significant failures in regulation and supervision. It has made the Financial Market Law and Compliance a key topic on the current agenda (Anagnostopoulos, 2018). However, Petrasic et al. (2016) maintain that, in 2014, the FSA provided the initial momentum initiating RegTech investment by ordering regulatory agencies with oversight of financial institutions to identify technologies supportive of compliance efforts to stymie money laundering.

Estimates of the amount of money laundered worldwide range from US$500 billion to US$1 trillion [MoneyVal (2017): 2]. Higher estimates of funds money laundered globally span 2–5% of global GDP amounting to US$800 billion-US$2 trillion annually [UNODC (2019)]. The ability to counter money laundering effectively remains challenged by a variety of factors. These include introduction of new and emerging threats (e.g. cyber-related financial crimes); gaps in regulatory regimes, including uneven availability of technical assistance for anti-money laundering purposes; and the costs associated with banks’ compliance with global anti-money laundering guidance (Miller and Rosen, 2017). Legal and regulatory frameworks undergirding anti-money laundering compliance have been strengthened over the years by applying stricter rules and regulations that comport with best practices and guidelines of the Financial Action Task Force, the global money laundering and terrorist financing watchdog, although effectiveness in suppressing money laundering generally lags behind technical compliance – and increasingly so [MONEYVAL (2017): 6]. Nonetheless, costs of non-compliance in the form of fines imposed on financial institutions are substantial with regulators around the world having imposed $26 billion in fines for non-compliance with anti-money laundering, know your customer (KYC) and sanction regulations in the last decade (Fenergo, 2018). In addition, criminal prosecution of Anti Money Laundering (AML) law violators have resulted in numerous, high profile cases ending with convictions [BTC-e (BitCoin Exchange) of major banks, among others, HSBC that was fined $1.93 billion by U.S. authorities (Viswanatha and Wolf, 2012).

Taking into account the necessities and the requirements as well as the possible negative consequences explained above, this paper is devoted to an in-depth study of bankers’ perceptions on these issues. Specifically, the main purpose of this study is to determine the impact of regulatory technology on money laundering prevention effectiveness in banks in the Arabian Gulf region using Bahrain as a case study of a regional banking hub. In the period 2012–2017, Bahrain conducted 43 investigations of money laundering resulting in ten convictions with terms of imprisonment of up to seven years and with fines of up to BHD200,000 [MENA-FATF (2018)]. This study subsequently explores the impact of regulatory technology on the ability of a financial institution (FI) to fight money laundering by addressing the following question: what is the overall effect of individual components of RegTech and electronic know your customer (eKYC) on money laundering prevention effectiveness? Based on a survey of 100 bankers in Bahrain, the results of multivariate analysis indicate that transactions monitoring through RegTech and cost- and time-saving aspects of RegTech, drive money laundering prevention effectiveness to a highly statistically significant extent. However, electronic know your customer (KYC) technologies are insignificant as drivers. These outcomes are the result of a study conducted for the first time in the Kingdom of Bahrain and illustrative of the Arabian Gulf region.

The paper is organized in the following manner: a literature review is followed by a section on methodology. Data analysis is further explained. In the final part, conclusions are put forth and a consideration of implications receives attention future studies are suggested.

## 2 Literature review

Complying with anti-money laundering rules, monitoring customer transactions, and undertaking customer onboarding necessary “to know your customer” is a very costly, complicated and time-consuming process. Although likely at the high end of estimates, U.S. financial services firms are estimated to spend over US25B annually on personnel and systems for AML compliance [KYC-360 (2018)]. However, rapid developments in RegTech are increasingly providing innovative compliance solutions in terms of monitoring, lowering cost, instituting better analysis and reducing associated risks [Special Report (2016)]. In that vein, Zabelina et al. (2018) define RegTech as set of regulatory technologies that helps organizations to comply constantly with the ever-evolving requirements of the law and promises financial institutions reliable, safe and economical solutions to increase their efficiency in that sphere, while Baxter (2016) defines RegTech as an application of technology to regulatory activities. RegTech offers innovative solutions involving automating money laundering prevention tasks involving multi-sourced collection of complex and fragmented data difficult to vet manually. In general, RegTech assists banks in meeting regulatory obligations by providing anti-money laundering risk data; customer onboarding, screening and monitoring for know your customer processes; and analytics of customer transactions [Comments of Grinblatt, Calvin and Ryan in FORUM: KYC technology for screening, verification and monitoring (2018)].

Kavuri et al. (2019) laments the dearth of literature on RegTech: “Although there is literature on Regtech, it is almost exclusively based [on legal issues].” Other studies focus on the technologies on a descriptive basis [Enriques (2017)] or a scientific-technical basis [Treleaven et al. (2017)]. Enriques (2017) focuses on the uses of RegTech from the perspective of regulators. Treleaven et al. (2017) explores automation using blockchain technology from the perspective of computer science. Financial Action Task Force, (2018) takes an in-between approach maintaining that legacy AML systems are being rapidly obsolete by a swath of emerging technologies - biometrics technology, blockchain, machine learning – combined with innovation in management information technology systems that will enable banks to visualize the behavior of their customers account activity in an era of multi-platform banking – a high risk money laundering environment.

There is a growing consensus on the importance and urgency for financial regulators to enhance their capacity using RegTech. RegTech is widely considered as holding a great potential to facilitate the supervisory process and enhance the regulatory compliance (Yang and Tsang, 2018). Studies looking into the efficacy of RegTech components as drivers of money laundering prevention effectiveness from alterative perspectives (economics/finance) are scant. Arner et al. (2015), although primarily viewing RegTech from a legal perspective, tangentially consider RegTech from the vantage-point of the effects on cost savings from digitization of manual reporting and compliance. While indubitably generating cost savings, the authors emphasize that the economic potential of RegTech vastly transcends cost-savings into transaction monitoring through pre-emptive risk identification tools designed to enhance the quality of compliance mechanisms. However, studies employing primary data used to make inference of the effect of RegTech on money laundering prevention effectiveness in the banking sector on the national level do not appear to exist – in the very least, no such studies have been identified by the researchers to exist. Accordingly, the current study will fill a massive lacuna in the literature.

Although no longer regionally preeminent, Bahrain remains a major financial center in the Middle East [Kerr (2018). Representing 16.5 % of GDP [Bahrain Economic Quarterly Q1 (2019), financial services constitute a key component of Bahrain's economy. The Central Bank of Bahrain (CBB), the regulatory authority of the Bahrain financial sector, accords “high priority” to protecting this sector from money laundering threats in its declaratory policy [CBB (2019)]. Money laundering archetypically proceeds through three stages: placement, layering and integration. Bahrain is more likely to be used in the layering stage of money laundering rather than in other stages [IMF Country Report (2007)]. Its relatively large financial sector with a network of foreign branches makes it particularly vulnerable to this risk (FATF, 2012). Overall, given its risk score (5.33) on the Basel AML Index 2018 above the 5.0 threshold, Bahrain, ranking in the middle (#65) out of 140 jurisdictions, can be adjudged as “having a significant risk of money laundering and terrorist financing” [MONEYVAL (2017): 4–5]. Hence, this study is vital to understand the capacities of a regional banking hub such as Bahrain. Bahrain is also an interesting case study as it follows a dual, i.e., conventional and Islamic banking practices. While conventional banking has been well studied with regards to anti-money laundering practices, the Islamic banking research lags behind and is often subject to biased perceptions. Nonetheless, some researchers stress that Islamic banking relies on a partnership with the customer, hence, such banks have to know their customers very well, as well as their business activities and sources and uses of funding [El Banna and Badr (2011)]. In this manner, by nature they pay more attention to KYC procedures. However, other researchers find this customer-bank relationship a risk to anti-money laundering practices. Since the bank is considered a partner, if its customer engages in money laundering, the institution will be compromised as well. Consequently, it may not have the incentive to report suspicious transactions [Kyriakos-Saad et al. (2016): 9]. In addition, the complexity of Islamic finance may be another concern for transparency of transactions and funds. Ultimately, the standards set by FATF do not take into account the nature of Islamic banking but were developed solely based on the practices of conventional banking.

## 3 Research methodology

### 3.1 Research design

This research aimed to find and analyze the relationship between independent variables embodying regulatory technology with a dependent variable (Money Laundering Prevention Effectiveness) in banks. This study utilizes descriptive statistics which involve, as a starting point, gathering data that describe phenomena under observation and then organizes, tabulates, depicts, and analyzes the data so collected [Glass and Hopkins (1984)]. Finally, multivariate statistical methods are utilized to test the hypotheses postulated in the research objectives with, ultimately, relevant conclusions and recommendations being drawn.

### 3.2 Population and sample size

Primary data has been elicited by questionnaire targeting bank employees because of their awareness and knowledge about money laundering prevention. As per CBB manpower survey [CBB (2018)], the banking sector, as of the end of the year 2017, attained a level of 7447 employees on the payroll in Bahrain. Staff in the banking sector are presumably educated and trained regularly to inculcate and to enhance skills in the combating of money laundering. Such actors are well-situated to gauge the impact of regulatory technology on money laundering prevention. The sample size for this study is 100 bank employees drawn from a cross-section of functions in the banking sector. This sample size generalizes over the population of 7, 447 banker employers of Bahrain; based on confidence level of 95%, and confidence interval of 9.73.

### 3.3 Survey instruments’ design

An online survey was designed using Google Docs and was distributed among bank employees. The questionnaire includes two parts: the first collects demographic information like gender, age, position and years of experience of the respondents who are working in banks. The second poses a series of structured question designed to elicit bankers’ perspectives on salient factors of regulatory technology that are affecting money laundering prevention. In this second part, a Likert scale was adopted ranging from number one representing “strongly disagree” to number five representing “strongly agree”.

### 3.4 Validity and reliability of the research instrument

The validity of the research instrument refers to the extent to which an instrument measures the factors under study. The questionnaire designed by the researcher was reviewed by a money laundering prevention specialist and approved by an academic professor to ensure its validity.

Reliability is the degree of consistency of a measure. As a gauge of reliability, the survey data was measured by employment of Cronbach Alpha, which is well-supported in applied statistics [De Souza et al. (2017)].

### 3.5 Measurement of variables

To measure the impact of regulatory technology on money laundering prevention effectiveness in banks, bank employees answered 20 questions related to the independent and dependent variables. The study identified three independent variables of “RegTech:” electronic know your customer, transaction monitoring, cost and time efficiencies embedded in RegTech. Likewise, the dependent variable money laundering prevention effectiveness was also measured by obtaining respondent perspectives.

### 3.6 Data collection and analytical methods

Descriptive statistics provide insights into the collected data focusing on means and standard deviations, as well as skewness and kurtosis, generated by responses per posited statement. After assessing the normality of the data, then taking care to remove outliers, multivariate statistics are employed culminating with hypothesis testing using Spearman's correlation and regression analysis.

## 4 Data analysis and findings

In addition to the descriptive analysis of the data collected from the sample, the researchers present output of multivariate statistical techniques with a view to addressing conclusively the hypotheses postulated. The following subsections demonstrate the results and analysis.

### 4.1 Testing normality and outlier anomalies

To gauge normality, descriptive statistics were used specifically inspecting output against specific criteria indicative of normalcy:

[1] Standard Deviation <1.5
[2] Skewness ≤ |±1.5|
[3] Kurtosis ≤ |±3|

Only in one instance is the criteria for normalcy violated with respect to kurtosis of the fourth Cost&Time efficiency variable by 3.145 > 3 but, with respect to the overall result, this slight violation is negligible in the overall context and can safely be overlooked in terms of the overall judgment that the data set is normal (see Table 1).

**Table 1:** Evaluation of normality using descriptive statistics (n = 100).

Name of Variable	Std. Deviation	Skewness		Kurtosis	
	Statistic	Statistic	Std. Error	Statistic	Std. Error
MLP1	.95325	-1.102	.241	1.409	.478
MLP2	.73416	-.908	.241	2.341	.478
MLP3	.96943	-.440	.241	-.176	.478
MLP4	.90314	-.992	.241	1.643	.478
MLP5	.86363	-1.038	.241	1.762	.478
TM6	.92660	-1.042	.241	1.460	.478
TM7	.87594	-1.051	.241	1.670	.478
TM8	.88192	-1.064	.241	1.964	.478
TM9	.86334	-.937	.241	1.464	.478
TM10	1.04447	-.673	.241	-.258	.478
CT11	.85729	-.722	.241	.602	.478
CT12	.81650	-1.023	.241	2.268	.478
CT13	.91954	-1.034	.241	1.626	.478
CT14	.70918	-1.028	.241	***3.145***	.478
CT15	.84154	-.497	.241	-.157	.478
eKYC1	1.09341	-.611	.241	-.049	.478
eKYC2	.95827	-1.064	.241	1.280	.478
eKYC3	.92217	-.891	.241	.429	.478
eKYC4	.98939	-.897	.241	.428	.478
eKYC5	1.05006	-.934	.241	.630	.478

Bold italic indicates deviation from normality.

### 4.2 Validity and reliability

Cronbach alpha method was used to measure the internal consistency of the participants’ responses. It is one of the most commonly used reliability coefficients (Hogan et al., 2000). The test results in Tables 2 and 3 shows that all alpha coefficient values are above 0.70, therefore, the questionnaire is considered to have adequate reliability inasmuch as Shavelson (2004) regards .70 < α, .80 as minimally acceptable. However, validity need be gauged by assessing the Item-to-Item/Item-to-Total output with criteria as follows: Item to item Correlation≥0.2 [Duncan et al. (2018)] and Item to total correlation ≥0.5 [Francis and White (2002) and Kim and Stoel (2004)].

**Table 2:** Cronbach alpha and item-to-item/item-to-total correlation.

	Item	Cronbach alpha	Item to item Correlation≥0.2		Item to total correlation ≥0.5		Remarks
			Min	Max	Min	Max	
1.	MLP1-MLP5	0.737	0.094	0.611	0.312	0.678	Two items MLP1 and MLP3 were found to cause problems in correlation. Recommended for deletion.
2	CT11-CT15	0.806	0.378	0.608	0.514	0.621	All items accepted
3	TM6-TM10	0.713	0.071	0.581	0.302	0.661	Two items TM6 and TM10 were found to cause problems in correlation. Recommended for deletion.
4	eKYC1-eKYC5	0.723	0.130	0.679	0.266	0.708	Two items eKYC1 and eKYC5 were found to cause problems in correlation. Recommended for deletion.

**Table 3:** Cronbach alpha and item-to-item/item-to-total result after deletions (n = 68).

Var Set	Item	Cronbach alpha	Item to item Correlation≥0.2		Item to total correlation ≥0.5		Remarks	Min Valid Sample Size∗
			Min	Max	Min	Max		
1	MLP2, MLP4, MPL5	0.752	0.227	0.679	0.634	0.725	3 Items Accepted	33
2	CT11-CT15	0.806	0.378	0.608	0.514	0.621	All items accepted	28
3	TM7-TM9	0.776	0.56	0.581	0.635	0.65	3 Items Accepted	28
4	eKYC2-eKYC4	0.829	0.508	0.679	0.64	0.773	3 Items Accepted	28

∗ Based on Sample Size Calculation Based on Formula by Bonett with α = .01, β = .9 (power 90%) referenced in Bujang et al. (2018).

Considering responses gleaned in reference to all question items in the survey instrument, it was observable that, while data is normally distributed at least where n = 68, item correlations were found problematic with respect to six question items in the survey: EKYC1/5, MLP1/3 and TM6/10.

After deleting items causing correlation problems, the following improvements are observable:

### 4.3 Descriptive analysis

#### 4.3.1 Descriptive analysis of demographic variables

This part of the research is intended to analyze the demographic data collected from the questionnaire's first section. The results obtained from 100 respondents are summarized in the below table by gender, age, experience and position, whereas Table 4 outlines the awareness of RegTech in Bahrain and the enforcement actions that were taken against banks as a result of compliance violations.

**Table 4:** Demographic data.

	Response Category	Frequency (N = 100)	Percentage
Gender	Male	62	62%
	Female	38	38%
Age	Under 30 years	37	37%
	30–40 years	50	50%
	41–50 years	12	12%
	Above 50 years	1	1%
Experience	Below 5 years	26	26%
	5–10 years	32	32%
	11–15 years	28	28%
	Above 15 years	14	14%
Position	Front Office Staff	28	28%
	Operations	16	16%
	Compliance/AML	36	36%
	Audit	5	5%
	Others	15	15%

As shown in Table 5 that presents the results of demographic data regarding gender, a total of 62% of the sample size were male (representative of most of the respondents) while female made up a minority of only 38%. Unsurprisingly, the result corresponds with CBB's manpower report of year 2017 [CBB (2018)] that indicates 4,910 males are working in banks, representing 66% of the total workforce and the remaining 34% are females. With respect to participants' age, (87%) are below the age 40. Results show that there are 50% of bankers aged between 30-40 years, 37% under 30 years, while 12% are between 41-50 years and only one participant is above 50 years. Such a demographic profile reflects a concerted effort by FIs to recruit ever younger employees better able to adapt to the rapid changes in financial services involving deployment of emerging technologies [Meyer-Chatfield Group (2018)]. In terms of experience, questionnaire respondents span 26% below 5 years of banking experience; 32%, between 5-10 years; 28%, between 11-15 years; and 14%, above 15 years of experience. Noticeably, survey data were almost collected in roughly equal tranches of staff exhibiting varied levels level of banking experience. However, 42% of respondents' manifest extensive professional experience (11 years or greater) in the bank sector. Providing a balanced cross-section of responses from bankers with varied experience profiles contributes effectively to the robustness and reliability of the research outcomes by eliciting a balance of varied bankers' perspectives toward RegTech. With respect to the job functions of bankers participating in the survey, the top two banking posts occupied by respondents constitute compliance/anti-money laundering officer (36%) and front office staff (28%). Other respondents indicated bailiwicks in operation and miscellaneous functions at 16% and 15%, respectively, with only 5% performing an audit role. Accordingly, more than one third of the participants are working directly in anti-money laundering field and above one quarter are performing front office activities which include interacting with customers and conducting know your customer requirements. Consequently, the study results credibly represent compliance specialists' views on RegTech impact on money laundering prevention and variety of opinions from other roles addressing the same.

**Table 5:** General data.

	Response category	Frequency (N = 100)	Percentage
RegTech Awareness	Yes	47	47%
	No	53	53%
Enforcement against Bank	Yes	25	25%
	No	75	75%

The results indicated a close to 50/50 split about RegTech awareness. Regardless of this finding, the term RegTech is disseminating rapidly nowadays in Bahrain's daily newspapers and regulators' publications as well as by word of mouth among bank employees. Furthermore, RegTech is well-known by anti-money laundering staff particularly.

Low cognizance among banking respondents vis-à-vis RegTech may be attributable to low incidence of enforcement actions, at least heretofore, against banks. Only one quarter of respondents confirmed that actions have been brought against their banks due to compliance violations concerning money laundering. In Bahrain, where the regulator maintains that a successful supervision over the banks activities requires stringent enforcements in cases of breaches in requirements imposed on FIs, the cost of non-compliance is set to increase [CBB (2019)] in tandem with the cost of non-compliance rapidly increasing globally.

#### 4.3.2 Descriptive analysis of technical variables

To gain insight into the dependent and independent variables, descriptive statistics are overviewed. The Tables 6, 7, 8 and 9 illustrate the percentages of each response to the statements, mean and standard deviation.

**Table 6:** Electronic Know Your Customer variable analysis.

Statements	Frequency %						
	Strongly Disagree (1)	Disagree (2)	Neutral (3)	Agree (4)	Strongly Agree (5)	Mean	Std. Deviation
1	6	8	29	36	21	3.58	1.093
2	3	4	17	45	31	3.97	0.958
3	1	5	17	38	39	4.09	0.922
4	2	7	17	40	34	3.97	0.989
5	5	6	20	44	25	3.78	1.05

**Statements:**

1- There are deficiencies in filling KYC form manually as some mandatory information may left out blank by client.

2- Automation that eliminates deficiencies in collecting required information from customer will strengthen the KYC process.

3- Obtaining customer data from government sources through automation can strengthen data reliability and verification requirements.

4- Updating KYC information electronically using government portal improve KYC process effectiveness.

5- Having inaccuracies in the information collected from customer may lead to your bank being used to launder funds and a regulatory penalty.

**Table 7:** Transaction Monitoring variable analysis.

Statements	Frequency %						
	Strongly Disagree (1)	Disagree (2)	Neutral (3)	Agree (4)	Strongly Agree (5)	Mean	Std. Deviation
1.	3	4	18	50	25	3.9	0.927
2	2	3	16	49	30	4.02	0.876
3	3	2	20	52	23	3.9	0.882
4	2	4	19	53	22	3.89	0.863
5	2	12	18	40	28	3.8	1.044

**Statements:**

1. Improved analytics for vast volumes of transactions to identify abnormal patterns can help detect suspicious activity more accurately.

2. Advanced system that checks accounts against watch-lists, screen transactions for sanctions can effectively help banks comply with economic sanctions.

3. Automation that enables access to authority's databases to conduct background screening/criminal record will enhance bank's AML risk assessment.

4. Improved data analysis that provides smart prediction & enable banks to visualize customer behavior will help banks to act proactively.

5. It's nearly impossible to monitor transactions without the support of an automated system.

**Table 8:** Cost and Time variable analysis.

Statements	Frequency %						
	Strongly Disagree (1)	Disagree (2)	Neutral (3)	Agree (4)	Strongly Agree (5)	Mean	Std. Deviation
1	1	7	20	53	19	3.82	0.857
2	2	1	18	53	26	4	0.816
3	4	3	23	52	18	3.77	0.92
4	1	1	11	60	27	4.11	0.709
5	0	8	21	51	20	3.83	0.842

**Statements:**

1. New Technologies (advanced software) can help banks cut the total cost of money laundering prevention.

2. Automated system for detecting suspicious activities that reduces false positive alerts will help AML specialist executing alerts in short time.

3. Automation submission of suspicious transaction reports enables authorities to receive reports in real-time.

4. Information collected in real-time by KYC automation reduces both cost and time.

5. The rapid changes in regulatory environments and fines imposed on banks causes the cost of money laundering prevention to increase.

**Table 9:** Money Laundering Prevention Effectiveness variable analysis.

Statements	Frequency %						
	Strongly Disagree (1)	Disagree (2)	Neutral (3)	Agree (4)	Strongly Agree (5)	Mean	Std. Deviation
1	3	4	16	46	31	3.98	0.953
2	1	1	14	57	27	4.08	0.734
3	2	10	29	40	19	3.64	0.969
4	3	1	22	46	28	3.95	0.903
5	2	2	17	48	31	4.04	0.864

**Statements:**

1- Bank account managers exhibit a high degree of effectiveness in implementing KYC safeguards.

2- Monitoring systems, usually detecting suspicious transactions in timely manner, serve as effective tools in the combating of money laundering in the bank.

3- Penalties and enforcement imposed on the bank in the context of punitive anti-money laundering actions undertaken by regulators are largely under control.

4- Advanced technology is effectively employed by the bank to stymie new emerging money laundering threats.

5- Money laundering prevention programs undertaken by the bank effectively guard the bank against regulatory and reputational risk.

The first independent variable presents the effectiveness of conducting know your customer requirements electronically. Five statements were set for the above variable and majority of opinions belong to agree with mean ranging from 3.58 to 4.09 and standard deviation between 0.922 and 1.093. A plurality of respondents agree (36%) that electronic know your customer bears on money laundering prevention effectiveness.

It is noted that the first statement "deficiencies in filling KYC form manually" and fifth statement "Inaccuracies in customer information may lead to money laundering and penalty" had the lowest mean (3.58 and 3.78, respectively) and highest standard deviation (1.093 and 1.05, respectively) which could indicate a lack of concurrence with respect to these statements. This may be interpreted as some participants being insufficiently aware of the effect of gaps in customer identification. Geister (2008) underscores that not having enough information or having inaccuracies in the information collected by the bank may create a domino effect that leads down a slippery slope ending in money laundering and subsequent non-compliance. A plurality of 36% and 44% register agreement, respectively, with the first and fifth statements with next highest opinions, with respect to the first statement, being neutral at 29%, and, with respect to the fifth statement, being strongly agree at 25%.

With respect to the third statement, the level of those strongly agreeing at 39% barely nudges out the level of those agreeing at 38% which revolves around strengthening customer data reliability and meeting verification requirements by obtaining information from government sources using automation. It scored the highest mean 4.09 and lowest standard deviation 0.922.

With a mean equal to 3.97 and standard deviation 0.989, the respondents also affirmed the effectiveness of updating customer information electronically by exploiting government portal as a modality to improve the KYC process to which a plurality of 40% agreed with a further 34% strongly agreeing.

Moreover, results indicate that 45% and 31% of the respondents agreed and strongly agreed, respectively, that efficiencies generated in collecting know your customer information by greater employment of automated systems should strengthen the KYC process. Such opinions are in line with Basel (2018) in documenting that RegTech holds potential to address effectively deficiencies in KYC implementation in banks. By way of comparison, as revealed by the Reuters (2017) survey, only 39% of American bankers acknowledge using automated systems for screening.

As illustrated above, five statements were designed to measure respondents' perspectives regarding the independent variable "transaction monitoring". Overall findings reveal that the standard deviations were below 1 except for the fifth statement. Such a result indicates a relatively greater level of consistency in responses with respect to transaction monitoring relative to those with regards to eKYC. Furthermore, a minimum mean of 3.80 was scored across all statements which made it clear that participants confirmed the importance of automation and advanced systems on monitoring banking transactions. This particular finding comports with that of Protiviti (2013), who accords importance to a well-designed transaction monitoring system as a vital AML mechanism designed to detect suspicious transactions and report them to regulatory authorities in real time.

The first observation focuses on detecting suspicious transactions accurately by analyzing vast volumes of transactions using improved analytics. With 75% of the respondents answering the first statement affirmatively, either agreeing or strongly agreeing, with a mean of 3.9 and a standard deviation of 0.927, consensus levels exceed those observed in the USA, where survey results confirmed by Reuters (2017) indicate that 64 % of respondents consistently monitor transactions using automated systems.

The second statement elicited insights from respondents concerning the efficacy of advanced checking systems in aiding banks to comply with economic sanctions. 79% of the respondents assented to the statement with 49% agreeing and 30% strongly agreeing. Responses registered, in comparison with those appertaining to the other four statements, the highest mean at 4.02 combined with the second lowest standard deviation at 0.876.

While 52% of banks employees agreed and 23% strongly agreed that risk assessment would be enhanced by automation plugged into access to governmental databases for criminal screening, 20% were agnostic – registering the highest level of neutrality across all five statements. The third statement obtained a mean of 3.9 with a standard deviation (0.882) lower than all but one of the standard deviations registered by the five statements appertaining to transaction monitoring.

About 75% of the respondents, having agreed and strongly agreed by 53% and 22%, respectively, that visualizing customer behavior and providing smart prediction would assist banks to act proactively, voiced their affirmation to the fourth statement. However, 19% are agnostic. That the statement attained a mean of 3.89 and a standard deviation of 0.863 engenders some support for the proposition that bankers are supportive of deployment of RegTech in this sphere.

The last statement putting under the spotlight respondents' views regarding the impossibility of monitoring transactions without automation registered the least assent but greatest inconsistency in reflection of the highest standard deviation of 1.044 combined with the lowest group mean of 3.8. Despite affirmation by 68% of the bankers, 14 % of the participants expressed their disagreement whereas 18 % remained neutral. Such dissent and agnosticism most likely stems from the level of experience (more than 11 years) in the banking industry of the dissenters and agnostics, who, for much of their career, were, it is reasonable to conjecture, accustomed to more manual approaches so as to evince a lesser propensity to adapt to technological shifts in methods of operations relative to younger cohorts.

In order to measure the last independent variable “cost and time,” five statements were used. All statements were confirmed by the respondents as the minimum mean value was 3.77 and the maximum standard deviation was 0.92 which is below 1, indicating a good consistency, in which majority of respondents assented to all statements, agreeing or strongly agreeing, with at levels of 70% at a minimum.

73% of the participants indicated their concurrence with the proposition concerning the potential of new technologies to reduce the cost of money laundering prevention in banks. Particularly, it was strongly agreed by 19%, agreed by 53% and about 20% were neutral. Also, the mean and standard deviation of the first statement were 3.82 and 0.857 respectively. These results confirm the strong belief that technology contributes efficaciously to cost reduction.

The acceptance level of the second statement linking reduction of false positives with time economy of bankers through automation was high with a mean of 4 and standard deviation of 0.816. That 53% of respondents agreed and 26% strongly agreed, manifests the consensus of bankers that RegTech solutions enhances efficiency by cutting the time needed to generate accurate alerts.

Similarly, majority of the respondents concurred with the third statement, with 52% agreeing and 18% strongly agreeing, concerning the efficacy of generating suspicious reports in real-time by using automation. However, 23% were neutral and 7% disagreed or strongly disagreed, indicative of a level of agnosticism or disagreement greater than that registered with respect to the other four statements. With a mean of 3.77 and standard deviation of 0.92, the results nonetheless lend credence to the statement that technology enhances the process of submission suspicious reports.

87% concurred with the fourth statement, with 60% agreeing and 27% strongly agreeing, concerning cost and time savings engendered by collecting KYC in real time through automation. It is important to note that responses to the statement registered the highest mean with 4.11 (indicative of the greatest level of concurrence) and the lowest standard deviation with 0.709 (indicative of the greatest level of consistency) which reflects the strong support of respondents for automating the KYC requirement to generate real-time information on customers.

The final statement addresses the linkage of cost of money laundering prevention with rapid regulatory changes and escalating fines for non-compliance. 71% affirmed the linkage, with 51% agreeing and 20% strongly agreeing – levels in excess of those observed in UK, where a reported 63% of surveyed respondents confirmed an increase in compliance cost associated with those factors that spurred U.K. banks to ratchet up investment in AML in terms of both staffing and technology (LexisNexis, 2017). A mean of 3.83 and standard deviation of 0.842 lends credence to the proposition that rapid regulatory changes and escalating fines for non-compliance increasingly render money laundering prevention protocols substantially more costly.

Table 4.8 illustrated the participants’ responses towards the dependent variable "money laundering prevention effectiveness” in banks. In general, the outcomes yielded means in the range of 3.64–4.04 (indicative of general confidence of respondents in AML effectiveness levels attained by their FIs) and standard deviations below 1 (indicative of consistency of judgment by respondents over all these section statements).

With 46% agreeing and 31% strongly agreeing, 77% expressed confidence in KYC effectiveness at their respective FIs. A mean of 3.98 and standard deviation of 0.953 supports the notion that bank managers are employing KYC systems as effective defenses against money laundering threats.

The second statement measured the effectiveness of monitoring systems that detect suspicious transaction in timely manner. 84% expressed their confidence, with 57% agreeing and 27% strongly agreeing, while only two bankers expressing reservations with respect to the effectiveness of monitoring systems at their respective banks. Given the highest mean of 4.08 and lowest standard deviation of 0.734 across the five statements, the confidence expressed can be plausibly deemed substantial and consistent.

The imposition of multiple enforcement actions on banks by regulatory authorities are surely a sign of failure in compliance. The third statement questioned the respondents if the penalties imposed on banks are under control as a result of prevention programs instituted at their banks. A mean of 3.64 and standard deviation 0.969 reflects lowest and most variable results of the five statements. The outcomes found that 40% of participants agreed and 19% strongly agreed, whereas 29% were neutral and 10% expressed their disagreement in terms of lack of control; hence, while a majority of banks have placed a lid on enforcement actions, a minority continues to incur penalties attributable to deficiencies extant in their anti-money laundering programs.

The fourth statement, eliciting responses concerning the impact of the effectiveness of advanced technology on combating emerging money laundering threats, generated a mean of 3.95 and a standard deviation of 0.903 with 46% of respondents agreeing and 28% strongly agreeing. The results lend credence to the proposition that banks are effectively employing advanced technology as an effective tool in combating money laundering.

In the final statement, with which the majority concur, with 48% agreeing and 31% strongly agreeing, evidence obtains that demonstrates that money laundering program protects banks effectively from reputational and regulatory risk. Given a mean of 4.04 and a standard deviation of 0.864, it appears that money laundering prevention programs instituted at banks have effectively mitigated, for the most part, regulatory and reputational risk.

In summary, these findings indicate that, while demonstrating effectiveness in KYC and transaction monitoring through, in particular, deployment of efficacious technology, not all banks, albeit a minority, have managed to contain punitive anti-money laundering actions undertaken by regulators against banks. Notwithstanding, bankers, in the main, expressed confidence in the ability of their respective FI's money laundering prevention programs to impart an effective layer of protection against regulatory and reputational risk.

### 4.4 Correlation matrix

Pearson correlation is commonly used to measure and describe the strength and relationship between continuous non-ranked variables with no requirement for normality. Table 12 depicts the relationship across the independent and dependent variables:

Results indicate high statistical significance on money laundering prevention effectiveness, on the one hand, from, in descending order, cost and time, transaction monitoring and, albeit substantially lower than the preceding two dependent variables, electronic KYC, on the other.

### 4.5 Regression model

In determining the impact of RegTech on money laundering prevention, the study applied multiple regression analysis in order to measure the nature of relationship between the variables.

The regression model can be elaborated as:

$$MLPE=\alpha +\beta_{1}X_{1}+\beta_{2}X_{2}+\beta_{3}X_{3}+\varepsilon$$

MLPE = Dependent variable, Money laundering prevention effectiveness
X1 = First independent variable, Electronic know your customer
X2 = Second independent variable, Transaction monitoring
X3 = Third independent variable, Cost and Time
α = constant value
β = Slope value of independent variable.
ε = Random error (Refer to Tables 10 and 11).

**Table 10:** Pearson correlation matrix (n = 68).

	Money Laundering Prevention Effectiveness	Electronic Know Your Customer	Transaction Monitoring	Cost and Time Efficiencies
Money Laundering Prevention	1.000			
Electronic Know Your Customer	0.329∗∗∗	1.000		
Transaction Monitoring	0.545∗∗∗	0.514∗∗∗	1.000	
Cost and Time Efficiencies	0.649∗∗∗	0.443∗∗∗	0.534∗∗∗	1.000

**Note**: Correlation is significant at 1%∗∗∗, 5%∗∗ and 10%∗.

**Table 11:** Model summary.

R	R^2^	Adjusted R^2^	Std. Error of the Estimate
.707	.500	.477	.38612

describes the percentage of the variance in the dependent variable (money laundering prevention effectiveness) that is anticipated by independent variables (RegTech factors).

Table 12 illustrates multiple regression results for the model presented in the study. The below tables contain F statistics that defines the overall significance of the model, p-value is the probability that can be used to decide whether the study is significant or not and R-square which is the proportion of variance of dependent variable explained by independent variables.

**Table 12:** Coefficient of determination.

Model	Unstandardized Coefficients		Standardized Coefficients	t	Sig.	VIF
	B	Std. Error	Beta			
(Constant)	0.636	0.444		1.434	0.157	--
EKYC (X_1_)	-0.004	0.087	-0.005	-0.045	0.964	--
TM (X_2_)	0.356	0.125	0.346	2.855	.006∗∗∗	1.185
CTE (X3)	0.521	0.126	0.451	4.139	.000∗∗∗	1.518

Note: Coefficient of Determination is significant at 1%∗∗∗, 5%∗∗ and 10%∗ -- = not relevant.

The independent variables in this research encompassing aspects of RegTech explain about 48% of the variability in relation to money laundering prevention effectiveness. Other factors that affect money laundering prevention effectiveness not covered in this study represent the balance of 52%. Such other factors include but are not limited to bank endogenous factors as goodness of corporate governance, extent of commitment of senior management to compliance efforts and level of staff expertise [Vaithilingam and Nair (2017); Said and Erlane (2013)].

In referring to Table 13, two of three independent variables indicate RegTech factors – Cost&Time Efficiencies and Transaction Monitoring – engender a highly significant contribution to money laundering prevention effectiveness (the dependent variable) as the significance level of both factors registered below 0.01: respectively, .000 < .01 and .006 < .01. However, the third independent variable electronic know your customer is not significant with .964 >> .05 level of significance. VIFs are reported for the two significant dependent variables (transaction monitoring/cost&time efficiencies). Reported values are <5 so are satisfactory evidencing absence of multicollinearlity [Jones (2018)].

**Table 13:** Anova of the regression.

Model	Sum of Squares	df	Mean Square	F	Sig.
Regression	9.548	3	3.183	21.348	.000
Residual	9.542	64	.149		
Total	19.090	67			

The model is statistically significance in determining how RegTech impacts the money laundering prevention at a significance level of 0.000.

Regression equation can be depicted as: MLP = 0.636 + 0.356X_2_ + 0.521X_3_ +ε.

#### 4.5.1 Testing hypothesis

H_0_ There is no significant impact of electronic know your customer provided by RegTech on money laundering prevention effectiveness

As shown in Table 13, the correlation value between eKYC and money laundering prevention is 0.329 at the 0.01 significance level, indicative of a low positive correlation between the variables. More critically, however, Table 13 shows that eKYC has no real impact on money laundering prevention given an absence of statistical significance even at the very lowest threshold of the latter (.964 >> .10) with a beta hovering around zero. This indicates that eKYC engenders no measurable impact on money laundering prevention effectiveness. Thus, the null hypothesis is accepted.

At first blush, this result seems counter-intuitive. One possible explanation is that bankers in Bahrain view non-electronic KYC mechanisms and modalities at their respective FIs as effective such that the increment to money laundering prevention effectiveness from upgrading to electronic KYC embedding advanced RegTech algorithms is, for the most part, deemed marginal with banks having already gone digital, on their recognizance. Banks in Bahrain already plug into a digital (national) ID system to authenticate their customers' identities and retrieve basic information accessed through CPR and CR numbers. So, through this extension of e-government, banks collect and verify a customer's identity electronically without physically collecting forms – a key element, according to Pisa and Woodsome (2019) of eKYC as a process in which banks improve the onboarding process by eliminating paper-based procedures and record-keeping, which results in reducing cost and time spent on verification – hence, more profitability for banks. Another explanation of the non-intuitive result engendered through the multivariate analysis may be that Bahraini bankers -- at least those whose functional specialization lies outside compliance/AML -- lack awareness of the disruptive impact of advanced technology like blockchain, identified by Lootsma (2017) on KYC effectiveness. In the sample, only 36% hold positions in compliance/AML.

H_0_ There is no significant impact of transaction monitoring provided by RegTech on money laundering prevention effectiveness

Table 13 illustrates the correlation value between transaction monitoring and money laundering prevention which is 0.545 at the .01 significance level. Results show a moderate positive correlation between the variables. In addition, Table 13 shows that advanced transaction monitoring solutions provided by RegTech have effect on money laundering prevention effectiveness to a likewise highly significant degree also at the 1% significance level. Given that results are highly statistically significant, the null hypothesis is accordingly rejected. The beta associated with transactions monitoring in the regression equation is substantial, though not overwhelming, at .356. Such results are intuitive. Transaction volumes have reached such magnitudes that it is widely conceded that RegTech holds its greatest promise in the sphere of transaction monitoring through such technologies as machine learning [FCA (2017)]. Anecdotally, in informal communications with compliance specialists employed in banks operating in Bahrain, the researchers have recorded statements to the effect that these bankers appreciate the potential of machine learning and data analytics as mechanisms to reduce the false positive alerts in transaction monitoring compounded, in the Middle East, by many alternate spellings of Arabic names in English. RegTech technologies such as machine learning update and collate data with a view to identification of risk in real-time while minimizing false positive alerts [Ball (2017)]. Transaction monitoring assists in detecting abnormalities in customer transactional behavior – a daunting task for large banks with the burden of screening millions of transactions on daily basis in a short time Balooni (2017). None other than the Basel Committee on Banking Supervision (2018) posits that RegTech solutions will engender efficiencies in money laundering prevention through the linkage of analytics of non-structured data with machine learning. Such linkages are anticipated to assist banks in monitoring the large volumes of customer transactions and in the reportage of suspicious transactions.

H_0_ There is no significant relationship between cost and time efficiencies built into RegTech and money laundering prevention efficiency

The relationship between cost and time using RegTech and money laundering prevention is displayed in Table 13. The correlation value of 0.649 at a .01 significance level evidences a strong correlation between the variables. Furthermore, Table 13 provides compelling evidence that RegTech can reduce the cost incurred by and time consumed in money laundering prevention activities substantially given RegTech solutions integrating automation, scalability, flexibility and security. Of betas associated with the dependent variables in the regression equation, the largest at .521 is attributable to cost and time at a significance level of .01. Accordingly, the null hypothesis is again rejected. This result comports with insights derived from the literature linking cost reduction to efficiencies in compliance processes instilled through adoption of RegTech [Deloitte (2017)] – especially early adoption thereof [Nieh (2017)]. As likewise observed by Elliot Burgess (2017), head of product and client services at JWG Group Ltd., the main benefit of adopting RegTech, follows from enabling banks to establish and maintain their controls cheaper, easier and faster [Tennant (2017)] as well as to conserve time through enabling banks to interpret vast quantities of data precisely in real-time. In a similar vein, the Basel Committee on Banking Supervision (2018) adamantly maintains that innovative technologies cost-effectively support bank regulatory requirements on anti-money laundering.

## 5 Discussion

The main objective of the study was to determine the impact of regulatory technology, if any, on money laundering prevention effectiveness in banks. For the purposes of this study, RegTech is encapsulated by three independent variables: electronic know your customer, advanced transaction monitoring, and cost and time efficiencies. Money laundering prevention effectiveness serves as the dependent variable regressed against the independent variables. All data was collected during March and April 2019 through surveying the expert knowledge of bankers in Bahrain through random sampling of the population (7447) of bankers in Bahrain. Of the sample size of 100 bank employees, 32 unreliable respondents were dropped before effectuating multivariate analysis (Pearson's correlation and regression), leaving a residual sample size of 68. Of the 20 statements composing the online questionnaire eliciting Likert responses, six, deemed to have generated inconsistent output, were removed after descriptive statistics were initially presented. In so doing, the data was demonstrated to be normally distributed bereft of outliers with the instrument deemed reliable and valid.

The descriptive results provide some insights into perceptions of the respondents. The majority agreed or strongly agreed with all the statements with respect to all the independent variables – eKYC, transaction monitoring, cost and time – being linked to effective compliance and AML practices. However, some nuances are discernible. With respect to KYC, greater importance is attached to linking up with government sources of information as well as to automation; less importance is attached to gaps attributable to manual data collection. While, with respect to transaction monitoring, participants confirmed the importance of such features as improved analytics and advanced checking on monitoring banking transactions, some modicum of scepticism was raised with respect to over-automating. With respect to minimizing cost and time, participants, with greater consistency relative to other independent variables, placed weight on employment of automation and deployment of new technologies as drivers of efficiency. Of all statements, the greatest assent and consistency was registered to the cost-and-time statement that “Information collected in real-time by KYC automation reduces both cost and time.” For the most part, bankers consider that their respective FIs have achieved a substantive degree of success in money laundering prevention effectiveness with the caveat that not all banks have achieved containment of enforcement costs and, indeed, ¼ of bankers admit that their FIs have been on the receiving end of punitive regulatory measure for non-compliance.

Multivariate analysis conducted was two-fold: Pearson's correlation, to measure the nature of relationship between the variables; subsequently, regression analysis was used to measure the impact of RegTech on money laundering prevention. The study findings can be summarized as follows: two independent variables of RegTech, advanced transaction monitoring and cost and time, serve as highly significant drivers at the 0.01 level of money laundering prevention effectiveness. Above all, cost and time have the highest level of impact on money laundering prevention effectiveness. RegTech's ability to process large data in real time which reduces costs and improves accuracy in the screening of large volumes of transactions, strengthens the impact of cost and time on money laundering effectiveness prevention. However, the study indicates that electronic know your customer provided by RegTech does *not* have a significant impact on money laundering prevention notwithstanding a moderate positive correlation between the variables. What follows is that compared to conventional banks, the fact that Islamic banks possess by nature better knowledge of their customers does not have any impact on identifying and preventing money laundering transactions.

The study recommends Regulator and Banks move beyond adoption of electronic know your customer (eKYC) solutions which, although in their infancy, are already being deployed in banks across Bahrain pursuant to a CBB mandate [EDBS/KH/C/19/2019] with the National Bank of Bahrain being the first retail bank to the National e-KYC platform [BizBahrain (2019)]. This recommendation is clearly in line with the implications of the study of Arner et al. (2017). To push forward progress in automating transaction monitoring engendering cost- and time-efficiencies, banks ought to deploy advanced AML analytical tools and early warning systems. Accordingly, it is probable that upfront costs associated with acquisition or utilization of RegTech platforms by FIs, in term of obviating net negative cash flows associated with future accidental non-compliance, may well represent a positive net present value investment. RegTech platforms should be effective to the extent that they detect non-compliance that would otherwise not have been detected (effectiveness criterion) or they detect non-compliance that would have been detected through other means but at higher cost (efficiency criterion).

## 6 Conclusion

RegTech is a newly emerging term and scholarly studies in the field are in their infancy. Very few studies, surveys and publications have been published about the impact of RegTech on money laundering prevention in banks. Of those, most have been legal or technical in nature. No previous studies, as far as the researchers are aware, have used primary data elicited from bankers to investigate the impact of RegTech on money laundering prevention in banks. For the Gulf Cooperation Council countries, certainly, this is a first. Insights drawn from this study in Bahrain will benefit regulatory authorities, financial institution, anti-money laundering specialists, bank employees and RegTech providers, in Bahrain and beyond, by gauging the efficacy of RegTech in combating money laundering.

A future study might repeat the current study but instead focusing on a smaller population: the banking staff at specifically denominated number of banks in Bahrain with a view to assessing the variance of responses across these banks. Future studies might also consider two other divergent lines of inquiry. One could be purely descriptive but on a “micro”-level exploring: Which companies are the leaders of RegTech? What are the names of their commercial solutions? How much do they charge for them? Which banks in Bahrain have adopted them? How do Bahrain bankers evaluate the effectiveness of such tools? Another study could focus on “success stories” of RegTech around the world linking specific RegTech solutions to real money laundering red flags, not false positives, that otherwise would have gone unnoticed by a FI. Secondary data could be integrated with primary through a case study of compliance at a Bahraini bank.

Finally, this research presents some limitations. Online links to the survey were made available to human resource officers at banks which agreed to participate in the research project. Human resource officers were given instructions on how to distribute randomly the surveys to bankers. However, the researchers cannot be certain that these points of distributions followed the correct modalities. Nor was any data available on any possible non-response bias. The survey relies on expert knowledge. However, in Part I of the survey it is revealed that just over half of respondents never heard of RegTech. It is not exactly clear how to interpret that. It may simply be that while they have information about RegTech, they are simply unfamiliar with the term. For obvious reasons, the research had to proceed on that latter assumption.

## Declarations

### Author contribution statement

A. Hamdan and A. Sarea: Performed the experiments; Contributed reagents, materials, analysis tools or data.

M. Turki: Conceived and designed the experiments; Analyzed and interpreted the data; Wrote the paper.

R.T. Cummings: Performed the experiments; Analyzed and interpreted the data; Wrote the paper.

M. Karolak and M. Anasweh: Contributed reagents, materials, analysis tools or data; Wrote the paper.

### Funding statement

This research did not receive any specific grant from funding agencies in the public, commercial, or not-for-profit sectors.

### Competing interest statement

The authors declare no conflict of interest.

### Additional information

No additional information is available for this paper.
